# TRPM7 Induces Tumorigenesis and Stemness Through Notch Activation in Glioma

**DOI:** 10.3389/fphar.2020.590723

**Published:** 2020-12-14

**Authors:** Jingwei Wan, Alyssa Aihui Guo, Pendelton King, Shanchun Guo, Talib Saafir, Yugang Jiang, Mingli Liu

**Affiliations:** ^1^ Department of Microbiology, Biochemistry and Immunology, Morehouse School of Medicine, Atlanta, GA, United States; ^2^ Department of Neurosurgery, The Second Xiangya Hospital, Central South University, Changsha, China; ^3^ University of South Carolina SOM Greenville, Greenville, SC, United States; ^4^ Department of Chemistry, Xavier University, New Orleans, LA, United States; ^5^ Neuroscience Institute, Morehouse School of Medicine, Atlanta, GA, United States

**Keywords:** TRPM7, glioma, CD133, Aldehyde dehydrogenase 1, molecular targets, cell cycle, apoptosis

## Abstract

We have reported that transient receptor potential melastatin-related 7 (TRPM7) regulates glioma stem cells (GSC) growth and proliferation through Notch, STAT3-ALDH1, and CD133 signaling pathways. In this study, we determined the major contributor(s) to TRPM7 mediated glioma stemness by further deciphering each individual Notch signaling. We first determined whether TRPM7 is an oncotarget in glioblastoma multiforme (GBM) using the Oncomine database. Next, we determined whether TRPM7 silencing by siRNA TRPM7 (siTRPM7) induces cell growth arrest or apoptosis to reduce glioma cell proliferation using cell cycle analysis and annexin V staining assay. We then examined the correlations between the expression of TRPM7 and Notch signaling activity as well as the expression of GSC markers CD133 and ALDH1 in GBM by downregulating TRPM7 through siTRPM7 or upregulating TRPM7 through overexpression of human TRPM7 (M7-wt). To distinguish the different function of channel and kinase domain of TRPM7, we further determined how the α-kinase-dead mutants of TRPM7 (α-kinase domain deleted/M7-DK and K1648R point mutation/M7-KR) affect Notch activities and CD133 and ALDH1 expression. Lastly, we determined the changes in TRPM7-mediated regulation of glioma cell growth/proliferation, cell cycle, and apoptosis by targeting Notch1. The Oncomine data revealed a significant increase in TRPM7 mRNA expression in anaplastic astrocytoma, diffuse astrocytoma, and GBM patients compared to that in normal brain tissues. TRPM7 silencing reduced glioma cell growth by inhibiting cell entry into S and G2/M phases and promoting cell apoptosis. TRPM7 expression in GBM cells was found to be positively correlated with Notch1 signaling activity and CD133 and ALDH1 expression; briefly, downregulation of TRPM7 by siTRPM7 decreased Notch1 signaling whereas upregulation of TRPM7 increased Notch1 signaling. Interestingly, kinase-inactive mutants (M7-DK and M7-KR) resulted in reduced activation of Notch1 signaling and decreased expression of CD133 and ALDH1 compared to that of wtTRPM7. Finally, targeting Notch1 effectively suppressed TRPM7-induced growth and proliferation of glioma cells through cell G1/S arrest and apoptotic induction. TRPM7 is responsible for sustained Notch1 signaling activation, enhanced expression of GSC markers CD133 and ALDH1, and regulation of glioma stemness, which contributes to malignant glioma cell growth and invasion.

## Introduction

GBMs are the most malignant tumors of the central nervous system (CNS) in humans and have an extremely poor prognosis (Sander et al., 2017). The current standard of care for GBM patients includes surgical resection followed by adjuvant radiation therapy and chemotherapy with temozolomide (TMZ), an oral alkylating agent. Chemotherapy with TMZ may suppress tumor growth for a certain period of time; however, invariable tumor recurrence remains virtually inevitable, and most patients ultimately succumb to the disease (Melamed et al., 2018). Accumulating evidence shows that the failure of glioblastoma to current chemo- and radiotherapies and the high tumor recurrence rate are attributed to the presence of a small subpopulation of glioma stem cells (GSC), which is characterized by their stem cell-like properties and aggressive behaviors (Maher et al., 2001). Although controversy remains on the details about the precise GSC identity (Dirks, 2010; Alifieris and Trafalis, 2015), the existence of GSC is widely accepted. GSCs are a small subset of CD133+, and ALDH1+ cells with self-renewal properties and are capable of initiating new tumors, contributing to glioma progression (Lucena-Cacace et al., 2019). CD133 + cells, characterized by high telomerase activities (a sign of stem cell activity) (Ludwig and Kornblum, 2017), have been used as a molecular biomarker for GSC (Cheng et al., 2009; Brown et al., 2017; Lu et al., 2018); the proportion of CD133 + cells is an independent risk factor for tumor growth and time to malignant progression (Zeppernick et al., 2008). Aldehyde dehydrogenase 1 (ALDH1), another functional marker of cancer stem cells (CSC) (Ludwig and Kornblum, 2017), is a cytosolic protein that oxidizes aldehydes to carboxylic acid, and its high activity may contribute to stem cell maintenance. ALDH1 expression in astrocytoma is correlated with a high WHO grade of gliomas and predicts a worse prognosis in glioma patients (Liu et al., 2012; Sullivan et al., 2017; Wang et al., 2017). Because GSCs share neural precursor markers with neural stem cells (NSCs), glioma's high heterogeneity is greatly attribute to the current treatment failures against malignant glioma (Vescovi et al., 2006; Alifieris and Trafalis, 2015).

Notch signaling is highly active in GSCs, inhibits differentiation, maintains stem-like properties, and, therefore, is responsible for glioblastoma tumorigenesis (Bazzoni and Bentivegna, 2019). The Notch system in vertebrates is comprised of four receptors (Notch1–Notch4) and at least five ligands from the families Delta and JAG/Serrate (DSL): JAG1, JAG2, Delta-like (Dll)-1, Dll-3, and Dll-4 (Artavanis-Tsakonas et al., 1999; Miele, 2006; Miele et al., 2006). Notch receptors are activated in gliomas, and their oncogenicity has been confirmed by gain- and loss-of-function studies *in vitro* and *in vivo* (Teodorczyk and Schmidt, 2014). Notch signaling is not only central to the normal development of the CNS, but plays important roles in the proliferation, differentiation, apoptosis, and regulation of GSC. Notch signaling is also involved in regulating responses to hypoxia and angiogenesis, which are typical features for tumors, specifically GBM (Hovinga et al., 2010; Qiang et al., 2012; Stockhausen et al., 2012).

TRPM7 is a non-specific divalent cation channel fused with a functional serine/threonine-protein kinase domain at its C-terminus (Gautier et al., 2016). Physiologically, the TRPM7 channel contributes to calcium, magnesium, and zinc homeostasis, cell survival, gastrulation (Gautier et al., 2016), thymopoiesis, and embryonic development (Jin et al., 2008; Jin et al., 2012; Duan et al., 2018). Pathologically, TRPM7 overexpression is involved in malignancies (Gautier et al., 2016), neuronal death (Asrar and Aarts, 2013), and cardio fibrosis (Yue et al., 2013). The mechanisms by which TRPM7 facilitates the viability of tumor cells vary. TRPM7 is found to interrupt the cell cycle distribution and cell apoptosis in breast and bladder cancer (Cao et al., 2016; Liu et al., 2020), and deregulates senescence in hepatocellular carcinoma (Voringer et al., 2019). The TRPM7 kinase domain is cleaved by caspases (Desai et al., 2012; Krapivinsky et al., 2014) and participates in Fas-induced apoptosis (Desai et al., 2012). Given its multifaced function, TRPM7 has been recognized to be a promising drug target. A set of small organic modulators of TRPM7 either activating or inhibiting the TRPM7 channel, or regulating the kinase activity have been identified, and these potential drugs could lay strong foundations for the development of high-affinity *in vivo* drugs targeting TRPM7 (Chubanov et al., 2017).

Our previous report showed that in human glioma cells, TRPM7 expression is upregulated, required for proliferation, migration, and invasion (Liu et al., 2014; Leng et al., 2015), and mediated by multiple mechanisms through Notch, STAT3-ALDH1, and CD133 signaling pathways. In the current study, we further determined the specific component of Notch signaling that is critical in response to altered TRPM7 expression and results in the expression of GSC markers and treatment failure of current chemo- and radiotherapies against glioma.

## Material and Methods

### Antibody and Reagents

The following primary antibodies were used in this study. Rabbit polyclonal anti-TRPM7 (cat no. ab232455) and rabbit polyclonal Notch3 (cat no. ab60087) were purchased from Abcam (Cambridge, MA). Mouse monoclonal Notch1 antibody (cat no. N6786) and rabbit polyclonal Hey2 antibody (cat no. PA5-67647) were purchased from Invitrogen (Waltham, MA). Rabbit monoclonal Notch2 (cat no. 5732), rabbit polyclonal Survivin (cat no. 71 G4B7), and mouse monoclonal anti-HA antibody (cat no. 2367) were purchased from Cell Signaling Technology (Danvers, MA). Rabbit polyclonal Notch4 antibody (cat no. 07-189) and anti-β-actin antibody (cat no. A3854) were purchased from Sigma-Aldrich (St. Louis, MO). Rabbit polyclonal CD133 antibody (cat no. NB120-16518) was purchased from Novus Biologicals (Centennial, CO). Rabbit polyclonal ALDH1 was purchased from GeneTex (cat no. GTX123973, Irvine, CA). All secondary antibodies used for Western blot were purchased from Calbiochem (La Jolla, CA).

### Plasmid and siRNA

The wild-type human TRPM7 (wtTRPM7) or constructs in which the α-kinase domain was deleted (Δkinase) or rendered inactive with a point mutation in the ATP binding site of the α-kinase domain (K1648R) were provided by Dr. Carsten Schmitz, University of Colorado, Denver, CO. All constructs (wtTRPM7, Δkinase, K1648R) were tagged with a hemagglutinin (HA) at the N-terminal. Control scrambled siRNA (On-TARGETplus Non-targeting siRNA, catalog no. D-001810-01-05) and ON-TARGETplus SMARTpool siRNA (Catalog no. L-005393-000005) targeting TRPM7 were purchased from Dharmacon (Lafayette, CO). Control scrambled siRNA (cat no. sc-37007), and siRNA targeting siNotch1 (cat no. sc-36095) were purchased from Santa Cruz Biotechnology (Santa Cruz, CA). The scrambled siRNAs, with no homology to any known sequence, were used as controls.

### Cell Culture

Human glioblastoma cell lines. A172, was obtained from ATCC (Manassas, VA, USA). Other glioma cell lines, U87MG, U373MG, and SNB19, were kindly provided by Dr. Yancey G. Gillespie at the University of Alabama at Birmingham (UAB), Birmingham, AL. Dr. Hui-Kuo Shu at Emory University, Atlanta, Georgia, kindly provided the human glioblastoma cell line SF767. All cells were cultured in Dulbecco's Modified Eagle's Medium (DMEM, Life Technologies, Waltham, MA) plus 10% fetal bovine serum (FBS), 50 units/ml penicillin, and 50 µg/ml streptomycin at 37°C. Human embryonic kidney (HEK293) cells, with inducible expression of human TRPM7 channels (HEK: TRPM7 cells), were cultured in minimal essential medium supplemented with 10% FBS and antibiotics. For TRPM7 induction, cells were treated with 1 µg/ml of tetracycline. PDX lines: Tumor tissue cubes stored at liquid nitrogen (provided by Yancey G. Gillespie at UAB) were implanted subcutaneously into the flanks of male or female 6–8 weeks old nude mice under anesthesia (ketamine/Xylazin 90/6 mg/kg Bw). Briefly, cryopreserved tumor tissues were thawed at 37°C and washed with PBS before subcutaneous implantation. To prepare single-cell suspension of viable tumor cells, the xenograft tumor tissues were harvested and minced with scalpel blades followed by passing through cell strainers. The cells were then grown in DMEM/F-12 media plus 10% FBS, 50 units/ml penicillin, and 50 µg/ml streptomycin for future use.

Enrichment for glioma stem cells: For tumorsphere culture, glioma cell lines cultured in growth media were grown to confluence, dissociated using 0.1% trypsin, and dispersed by pipetting with a 23-gauge needle. After checking for a single cell, the cells were pelleted and suspended in sphere enrichment medium, specifically in human neurobasal medium supplemented with B27, 20 ng/ml EGF and 20 ng/ml FGF-2 (Invitrogen, Carlsbad, CA), and 5 μg/ml heparin (Sigma-Aldrich, St. Louis, MO). These cells were then plated in ultralow attachment surface tissue culture plates (Corning). Following overnight incubation at 37°C with 5% CO_2_, the distinct non-adherent human glioma stem cells were apparent in culture. These spheres were collected, gently centrifuged at low speed (1,000 rpm), passaged, and maintained for growth in the sphere enrichment medium for future use.

### Transfection of siRNA and DNA Constructs

When the glioblastoma cells grew to reach about 50–75% confluency, the appropriate amount of siRNAs specific to TRPM7, Notch1, and control scrambled siRNA with a final concentration of 100 nM, were transfected using Lipofectamine RNAiMAX reagent in serum-free OptiMEM-1 medium (Invitrogen, Carlsbad, CA) according to the manufacturer's instruction. After 6 h of transfection, cells were grown in DMEM containing 10% FBS further for 72 h as indicated in each experiment. 48 or 72 h post-transfection target knockdowns were assessed by RT-PCR or Western blot, respectively. Various glioma cells at 50–75% confluency were transfected with a pcDNA4/TO plasmid that allowed protein expression of wt hTRPM7 or hTRPM7 α-kinase inactive mutants by lipofectamine 3,000 transfection reagent (Invitrogen, Carlsbad, CA) according to the manufacturer's instruction. The transiently transfected glioma cells expressing wt hTRPM7 (M7-wt), Δkinase (M7-DK), and K164R hTRPM7 (M7-KR) constructs were maintained in DMEM containing 10% FBS (Schmitz et al., 2003; Perraud et al., 2011) for further growth for 72 h. The overexpression of TRPM7 and its mutants was assessed by HA expression. All studies were done in triplicates.

### MTT Assay

All glioma cells were seeded at 1 × 10^4^ cells in 100 μl of medium per well into 96-well plates and were transfected with 100 nM specific siRNA or control using Lipofectamine reagents for the indicated times. 10 μl of 3-(4,5-dimethylthiazol-2-yl)-2,5-diphenyltetrazolium bromide (MTT) reagent (Sigma-Aldrich, St. Louis, MO, the ratio of MTT reagent to the medium is 1:10) was added into each well and incubated in the dark at 37°C for 2–4 h. Absorbance at 570 nm was measured using 690 nm as the reference using the CytoFluorTM 2300 plate reader.

### Western Blotting

Cells were lysed with lysis buffer (50 mM HEPES, 150 mM NaCl, 1.5 mM MgCl2, 1 mM EGTA, 10% glycerol, 1% Nonidet P-40, 100 mM NaF, 10 mM sodium pyrophosphate, 0.2 mM sodium orthovanadate, 1 mM phenylmethylsulfonyl fluoride, 10 µg/ml aprotinin, and 10 µg/ml leupeptin). SDS/PAGE separated samples, and separated proteins were transferred to nitrocellulose membranes and identified by immunoblotting. Primary antibodies were obtained from commercial sources and were diluted at the ratio of 1:1,000 according to manufacturer's instruction. Blots were developed with Supersignal Pico or Femto substrate (Pierce). Densitometric analysis of the bands was performed with the ImageQuant program (Bio-Rad).

### Flow Cytometry

Cell cycle analysis and apoptosis assay: 1 × 10^6^ cells were harvested, fixed in ice-cold 70% ethanol, and resuspended in PBS for 1 min. After centrifuge at 450 × g for 5 min with the brake on low at room temperature, the cells were resuspended in 200 µl Guava Cell Cycle Reagent (cat no. 4500-0220, Luminex, Austin, TX) and incubated at room temperature for 30 min while shielded from the light. All samples were transferred to 96-well microplate plates with round bottom and acquired on a Guava easyCyte 8HT Base System (Luminex). The percentage of cells in G0/G1, S, and G2/M phases was determined from the DNA content using guavaSoft 3.1.1. The apoptotic glioma cells were detected by flow cytometry using Annexin V-PE and 7-AAD. The staining procedure was conducted with a Guava Nexin Reagent kit (cat no. 4500-0455, Luminex) according to the manufacturer'|’s protocol. Briefly, after desired treatments, cells were collected and resuspended in 100 µl of 1% FBS (Cell concentration should be between 2 × 10^5^ and 1 × 10^6^ cell/ml) followed by incubation with the 100 µl of Guava Nexin Reagent for 20 min at room temperature in the dark. The samples were then acquired on a Guava easyCyte 8HT Base System, which was used to detect apoptotic cells. Data were analyzed using InCyte software. CD133: To evaluate CD133 expression by flow cytometry, cells were harvested, washed with Cell Staining Buffer (cat no. 420201, Biolegend, San Diego, CA), and then incubated with PE-anti-human CD133 antibody (cat no. 372803, Biolegend, San Diego, CA) for 15–20 min on ice in the dark. Cells were then washed and suspended in Cell Staining Buffer for analysis. The data acquired on a Guava easyCyte 8HT Base System were analyzed using the InCyte software. Aldefluor assay: ALDH1 enzymatic activity was measured using the Aldefluor kit (cat no. 01700, Stem Cell Technologies). Cells suspended in the aldefluor assay buffer were incubated with ALDH enzyme substrate, BODIPY-aminoacetaldehyde (BAAA), for 30–60 min at 37°C. As a control for baseline fluorescence, cells were also treated with the ALDH inhibitor, diethylaminobenzaldehyde (DEAB). Fluorescence was detected using a Guava easyCyte 8HT Base System and analyzed using the InCyte software. Statistical significance was determined by the Student's test or one-way ANOVA tests.

### Bioinformatics Analysis

The expression of TRPM7 transcript in brain normal and tumor tissues were obtained from Sun brain samples from Oncomine (https://www.oncomine.org/). Statistical analysis of differences was performed using Oncomine algorithms for accounting the multiple comparisons among different studies similar to a meta-analysis.

### Statistical Analysis

The results obtained in this work were expressed as mean ± S.D. of at least three independent experiments done in triplicate. Paired Student t-test or one-way ANOVA tests were performed for data analysis, and a significant difference was defined as *p* < 0.05.

## Results

### Targeting Transient Receptor Potential Melastatin-Related 7, an Oncotarget in Glioblastoma Multiforme, Suppresses the Growth and Proliferation Through the G1/S Arrest of the Cell Cycle and Promotes the Induction of Apoptosis of Glioma Cells

**FIGURE 1:**
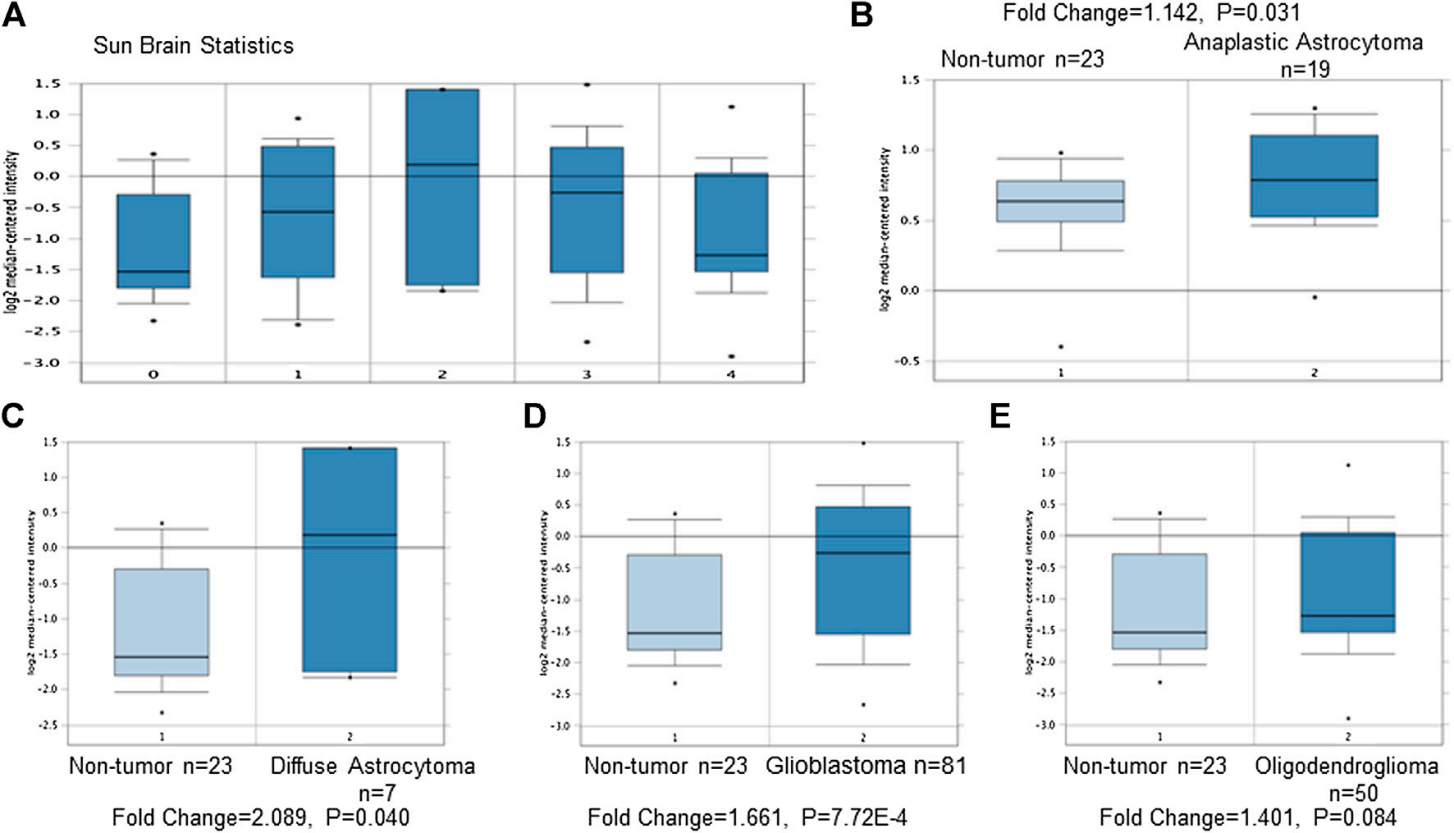
TRPM7 expression increases in glioma patients compared to that of healthy brain tissues. Meta-analysis was performed using the Oncomine Research Platform-based microarray studies. **(A)** A general overview of microarray analysis of normal brain tissues (0) and anaplastic astrocytoma (1), diffuse astrocytoma (2), glioblastoma (3), and oligodendroglioma (4). **(B–D)** In detail, microarray analysis of 23 normal brain tissues and 19 anaplastic astrocytomas **(B)**, seven diffuse astrocytomas **(C)**, 81 glioblastomas **(D)**, and 50 oligodendrogliomas **(E)**.

**FIGURE 2:**
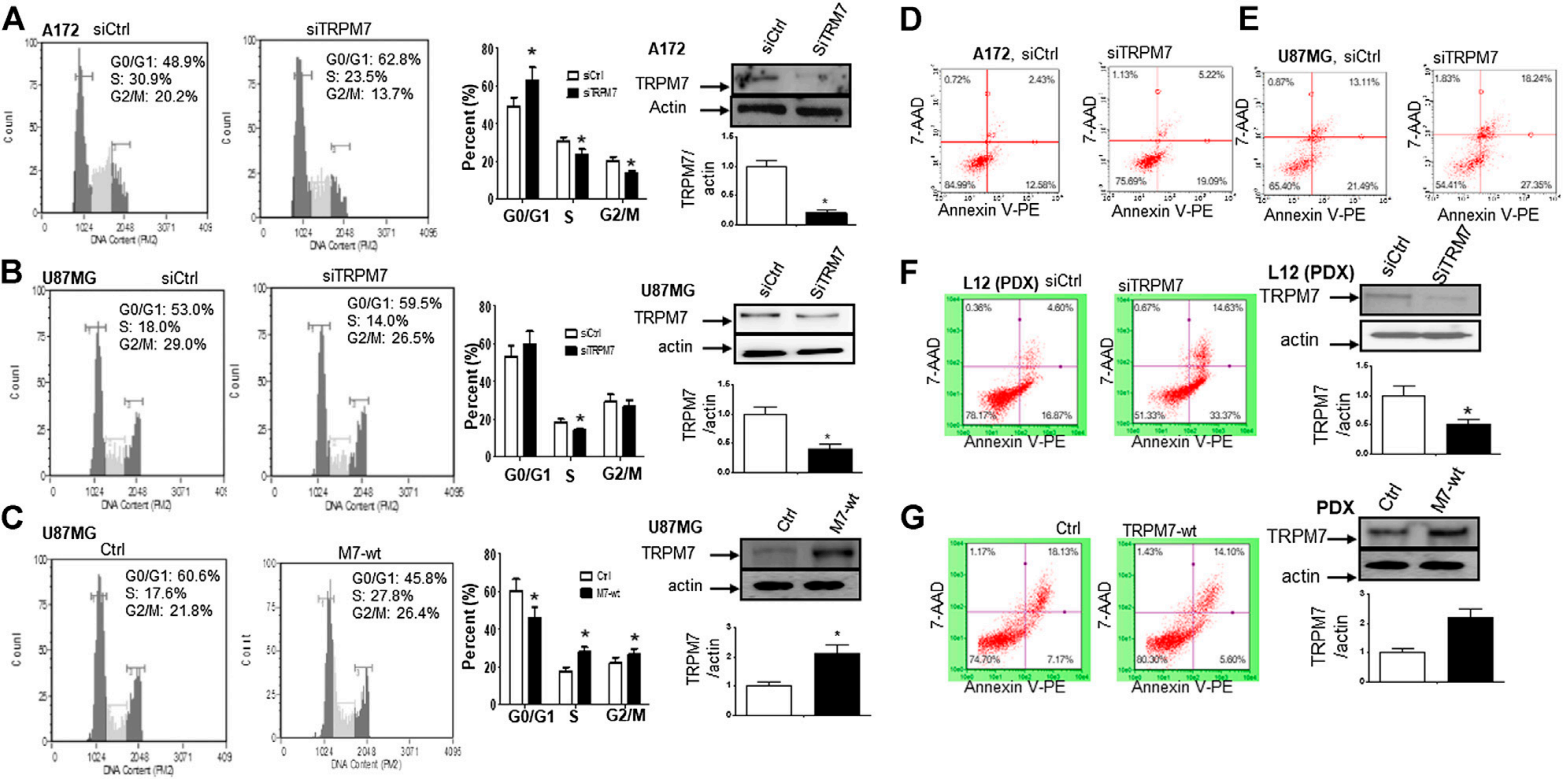
Changes in TRPM7 expression affects cell cycle progression and apoptosis in glioma cells. **(A and B)** TRPM7 silencing induces G0/G1 cell cycle arrest. Cell cycle progression was analyzed by flow cytometry. Representative histograms of cell cycle progression and bar graphs show the mean percentage of cells in G0/G1, S and G2/M phases of A172 **(A)** and U87MG **(B)** cells. The transfection efficiency was detected by the expression of TRPM7 protein, as examined by Western blot. Under the image is the corresponding densitometry analysis for Western blot performed using the ImageQuant program (rightmost panel). **(C)** Overexpression of TRPM7 compromised the G0/G1 cell cycle accumulation of U87MG cells. The transfection efficiency was detected by the expression of TRPM7 protein, as examined by Western blot. Under the image is the corresponding densitometry analysis for Western blot performed using the ImageQuant program (rightmost panel). **(D–F)** Knockdown of TRPM7 induced apoptosis. Apoptosis was analyzed by flow cytometric analysis of annexin V-PE staining. The percentage of apoptotic cells was significantly increased after siTRPM7 treatment in glioma cells A172 **(D)**, U87MG **(E)**, and PDX-L12 **(F)** cells. **(G)** Overexpression of TRPM7 decreases apoptotic cell death of PDX-L12 cells. The transfection efficiency was detected by the expression of TRPM7 protein, as examined by Western blot. Under the image is the corresponding densitometry analysis for Western blot performed using the ImageQuant program. The bar graphs indicate the mean ± S.D. of three independent experiments. All data represent a representative experiment from three independent experiments.

(1) TRPM7 mRNA is highly expressed in GBM patients compared to that in normal controls, which indicates that TRPM7 is an oncotarget in GBM. TRPM7 is ubiquitously expressed in all mammalian cells (Penner and Fleig, 2007; Bates-Withers et al., 2011; Runnels, 2011; Paravicini et al., 2012) and is necessary for cell survival, growth, and migration (Jin et al., 2012). Change in TRPM7 expression has been associated to cancers. (Zhou et al., 2014; Yee, 2017). Increasing evidence shows that TRPM7 plays a critical role in tumor cell proliferation and invasion (Prevarskaya et al., 2010), suggesting that TRPM7 could be a therapeutic target in human malignancies (Kim et al., 2008; Yee et al., 2012; Chen et al., 2015; Huang et al., 2017; Rybarczyk et al., 2017). Among brain tumors, our research group pioneered TRPM7's oncogenic function in glioma proliferation, invasion (Wan et al., 2019), and glioma stemness (Liu et al., 2014). To further explore whether or not TRPM7 can be a potential drug target in malignant glioma, we analyzed TRPM7 expression in publicly available glioma microarray studies using the Oncomine database and gene microarray data analysis tool (Rhodes et al., 2004a; Rhodes et al., 2004b). A meta-analysis of microarray gene expression data sets related to human cancer genes revealed that TRPM7 mRNA is highly expressed in glioma patients' tumor tissues compared to that in normal brain tissues (Figure 1A). Meta-analysis from published database demonstrated that TRPM7 expression is significantly increased in anaplastic astrocytoma patients (*t*-test: 1.928; *p*-value: 0.031; n = 19; Figure 1B), diffuse astrocytoma patients (*t*-test: 2.011; *p*-value: 0.040; n = 7; Figure 1C), and glioblastoma patients (*t*-test: 3.368; P-value: 7.72E-4; n = 8; Figure 1D) compared to that of normal brain controls (n = 23). Fifty cases of oligodendroglioma patients were also evaluated using the same Oncomine research platform, and the results showed that TRPM7 mRNA expression is increased but did not reach statistical difference compared to that of the control (*t*-test: 1.401; P-value: 0.084; n = 50; Figure 1E). These data indicate that TRPM7 mRNA is upregulated in glioma, including GBM patients, and suggests that TRPM7 is an oncotarget in GBM patients.
(2) Targeting TRPM7 suppresses the growth and proliferation of glioma cells through G1/S arrest and the induction of apoptosis. Our previous studies revealed that TRPM7 plays a vital role in glioma cell proliferation (Liu et al., 2014; Wan et al., 2019). To gain further insight into the role of TRPM7 in supporting proliferation, we then investigated whether or not the inhibition of cell proliferation by small interfering RNA against TRPM7 (siTRPM7) could deregulate cell cycle distribution. A172 and U87MG cells were treated with 100 nM of siTRPM7 and corresponding controls for 72 h, followed by flow cytometric cell cycle analysis. The percentage of cells in G0/G1, S, and G2/M phases was determined based on DNA content. The representative histograms and bar graphs of Figure 2A show that siTRPM7 significantly changed cell distribution of the cell cycle and is reflected by decreased percentage of cells in S phase (30.9–23.5%) and G2/M phase cells (20.2–13.7%) and concomitantly increased percentage of cells in G0/G1 phase (48.9–62.8%) compared to that of the control. The undetectable TRPM7 protein by Western blot provide confirmative evidence that TRPM7 was effectively silenced by siTRPM7 (Figure 2A, rightmost panel). Similarly, in U87MG cells, siTRPM7 treatment significantly decreased the percentage of cells in S phase from 18.0 to 14.0% and G2/M phase from 29.0 to 26.5%, while increased G0/G1 phase cells from 53.0 to 59.5% (Figure 2B, the histograms and the bar graphs). The transfection efficiency of siTRPM7 into U87MG is shown on the rightmost panel of Figure 2B. In parallel, we transfected 5 µg of wild-type TRPM7 constructs (M7-wt) into U87MG cells for 72 h to increase the expression levels of TRPM7. As a result, overexpressed TRPM7 significantly enhanced the percentage of cells in S phase from 17.6 to 27.8% and in G2/M phase from 21.8 to 26.4%, as well as decreased G0/G1 phase cells from 60.6 to 45.8% (Figure 2C, histograms and bar graphs). The high transfection efficiency was confirmed by increased TRPM7 protein expression detected by Western blot (Figure 2C, rightmost panel). The results indicate that TRPM7 downregulation causes an accumulation of GBM cells in the G0/G1 phase of the cell cycle, and TRPM7 overexpression specifically compromised G0/G1 cell cycle accumulation. Therefore, the functional studies using flow cytometry suggest that TRPM7 is an oncotarget in GBM as well. TRPM7 knockdown-induced suppression of the growth and proliferation of glioma cells could be through G1/S arrest and the inhibition of the tumor cell entry into S and G2/M phase.

The balance between the factors of cell division, cell cycle arrest, differentiation, and apoptosis determines the potential of cell growth and proliferation. Anti-apoptosis is a common mechanism of tumor progression. To further investigate the underlying mechanism in glioma cells with TRPM7 knockdown by TRPM7 siRNA that undergoes a failure of growth, we determined if the apoptotic process is involved in GBM cells when treated with TRPM7 siRNA and employed in an apoptosis assay. Briefly, glioma cells transfected with siTRPM7 for 72 h were stained with annexin V and 7-AAD and subjected to Annexin-FACS to detect apoptotic cell death. The degree of early and late apoptosis was detected as the percentage of cells positive for annexin V-PE without or with 7-AAD staining, respectively. Flow cytometric dot plots of A172 and U87MG cell treated with siTRPM7 displayed increased rates of annexin V positive cells, for A172, from 12.58 to 19.09 and 2.43–5.22%, respectively (Figure 2D); for U87MG, from 21.49 to 27.35, and 13.11–18.24%, respectively (Figure 2E).

The genetic difference of *in vitro* cell lines only partially mirrors the diversity of individual patient tumors. Due to the limitation of cell lines, the patient-derived xenolines (PDXs) have been widely used because they closely resemble the patient tumors from which they were acquired. PDXs are characterized and classified into four GBM molecular subtypes: Neural, Proneural, Classical, and Mesenchymal. We, therefore, determined whether or not silencing of TRPM7-mediated apoptosis in glioma cell lines can be phenocopied to the PDX model. To this end, the cells isolated from animals with amplified glioma Neural subtype (L12) were grown in regular growth medium and then transfected with either siTRPM7 to knock down or M7-wt to overexpress the TRPM7 protein. The results clearly show similar patterns as those in A172 and U87MG. The early and late apoptotic cells were increased from 16.87 to 33.37 and 4.60–14.63%, respectively, by silencing TRPM7 (Figure 2F). The reduced TRPM7 protein expression detected by Western blot on the right panel of Figure 2F confirmed the high transfection efficiency in these cells. Overexpression of TRPM7 resulted in a remarkable decreased late apoptotic cell from 18.13 to 14.10%, while early-stage apoptotic cells slightly decreased from 7.17 to 5.6%. However, it did not reach a significant difference (Figure 2G, the right panel demonstrates that M7-wt significantly increases TRPM7 expression). Our data suggest that the downregulation of TRPM7 suppresses glioma proliferation by G0/G1 phase arrest concomitant with apoptosis induction.

### Molecular Studies Indicate That Transient Receptor Potential Melastatin-Related 7 Is Expressed in Glioblastoma Multiforme and Correlated with the Activation of Notch 1 and Stemness

**FIGURE 3:**
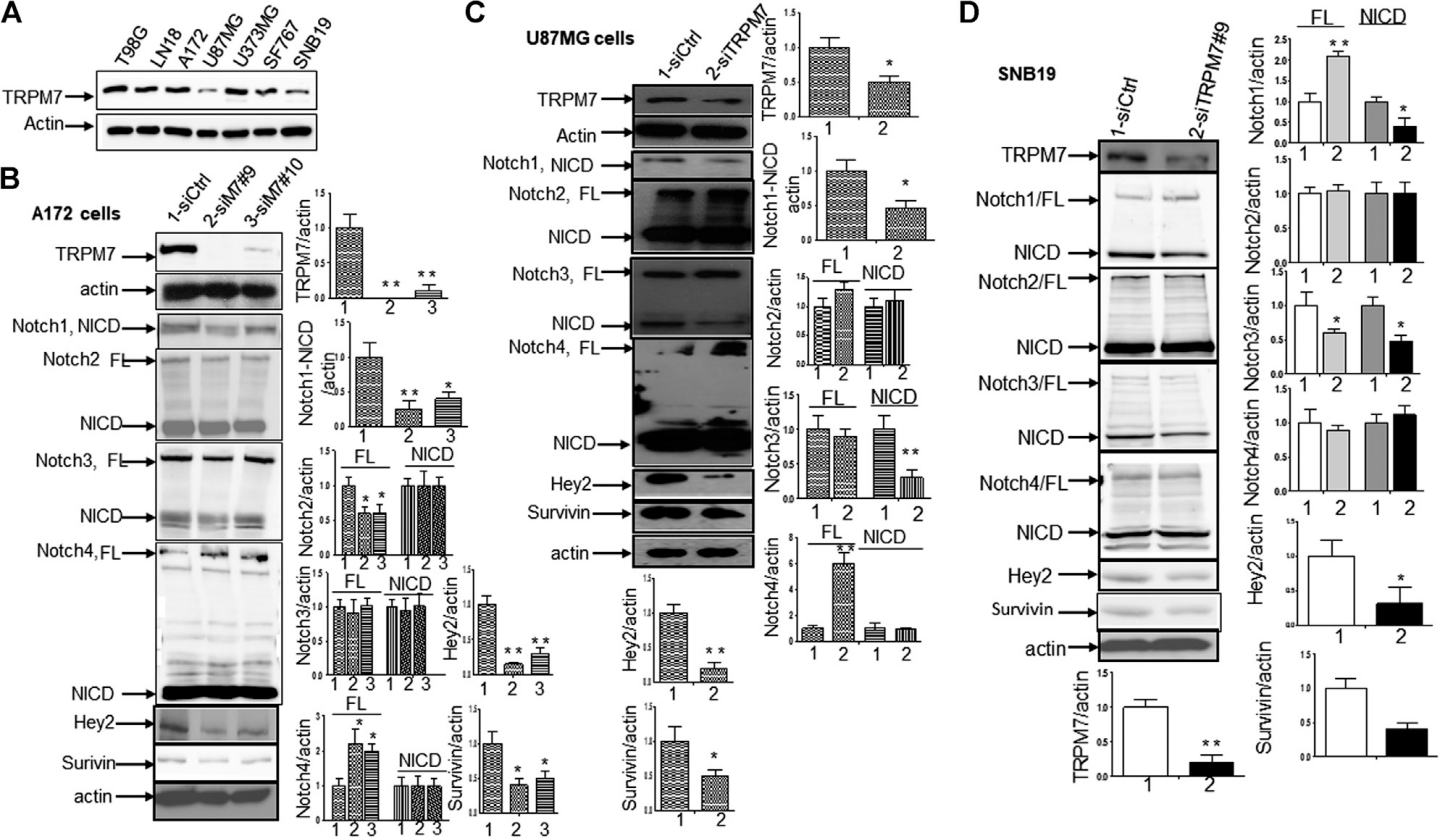
TRPM7 is expressed in GBM and positively correlates with the activation of Notch1, and the downregulation of TRPM7 inactivates Notch1 signaling. **(A)** The endogenous TRPM7 protein expression level was examined in all of the glioma cell lines by Western blot. **(B–D)** The glioma cells were transfected with siTRPM7 and control followed by assaying protein expression of TRPM7, Notch1, Notch2, Notch3, Notch4, and Notch target genes Hey2 and Survivin by Western blot in A172 **(B)**, U87MG **(C)**, and SNB19 **(D)** cells. To the right of each image is the corresponding densitometry analysis for Western blot performed using the ImageQuant program. The bar graphs indicate the mean ± S.D. of three independent experiments.

**FIGURE 4:**
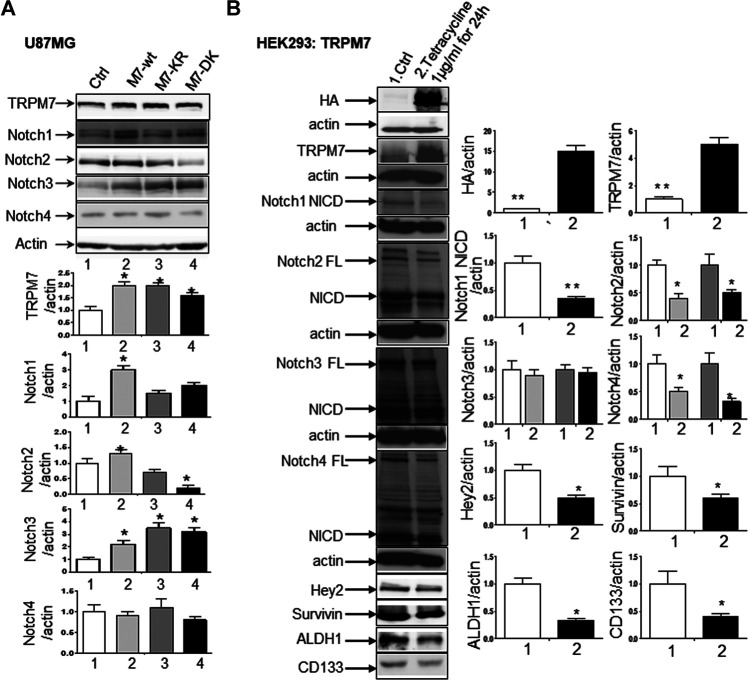
TRPM7 is expressed in GBM and positively correlates with the activation of Notch1, and the upregulation of TRPM7 activates Notch1 signaling. **(A)** The U87MG cells were transfected with **(A)** wild-type human TRPM7 (M7-wt); **(B)** two α-kinase-inactive mutants, “α-kinase-dead” point mutation (K1648R, or M7-KR) and α-kinase deleted mutant (M7-DK) along with controls followed by assaying protein expression of TRPM7, Notch1, Notch2, Notch3, and Notch4 by Western blot. **(B)** HEK-293 cells were transfected with a pcDNA4/TO plasmid that allowed tetracycline-inducible protein expression of TRPM7-wt tagged with HA. Then, protein expression of exogenous, endogenous TRPM7, Notch1, Notch2, Notch3, Notch4, and Notch target genes Hey2 and Survivin were determined by Western blot.

**FIGURE 5:**
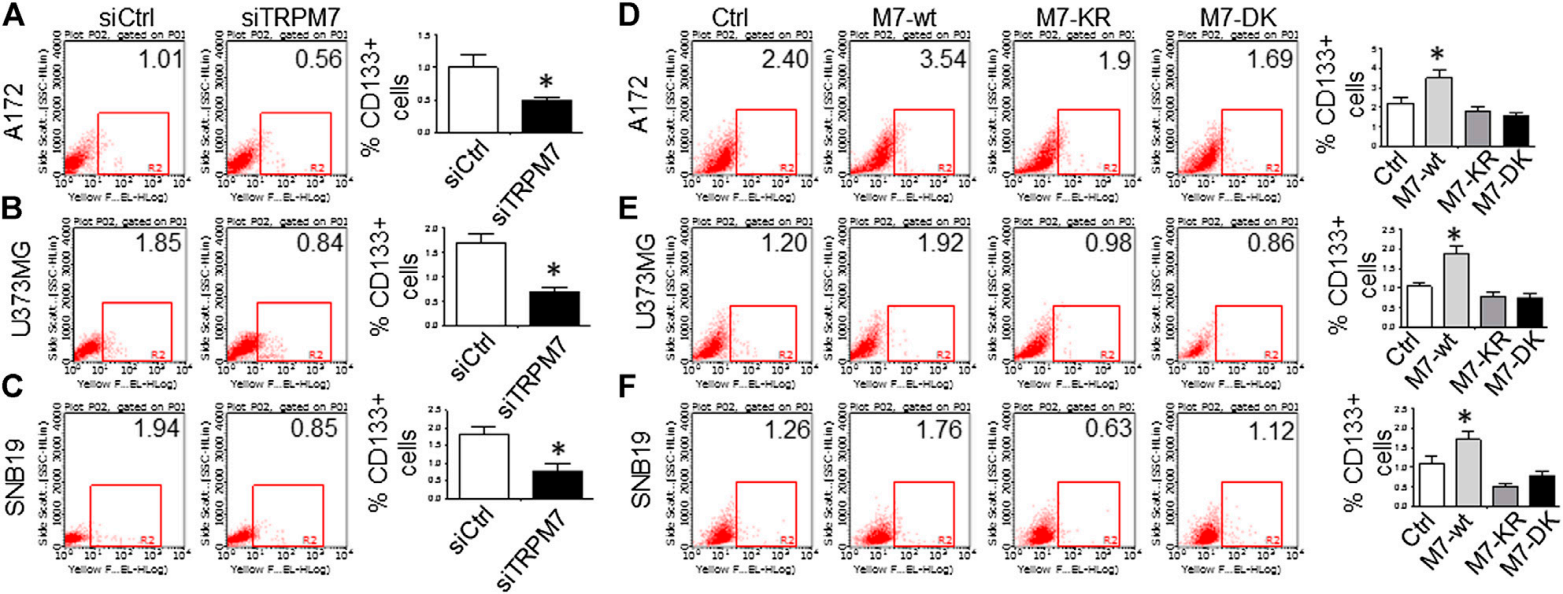
GSC marker CD133 is positively correlated with TRPM7 expression in glioma cells. **(A–C)** Flow cytometric analysis to measure CD133 expression was performed in A172 **(A)**, U373MG **(B)**, and SNB19 **(C)** glioma cells with TRPM7 silencing. **(D–F)** Flow cytometric analysis to measure CD133 expression was performed in A172 **(D)**, U373MG **(E)**, and SNB19 **(F)** glioma cells transfected with wild-type human TRPM7 (M7-wt) and two α-kinase-inactive mutants (M7-KR or M7-DK) along with controls. Bars on the right of each image represent the mean ± S.D. after normalization to control. All results are representative of three separate experiments.

**FIGURE 6:**
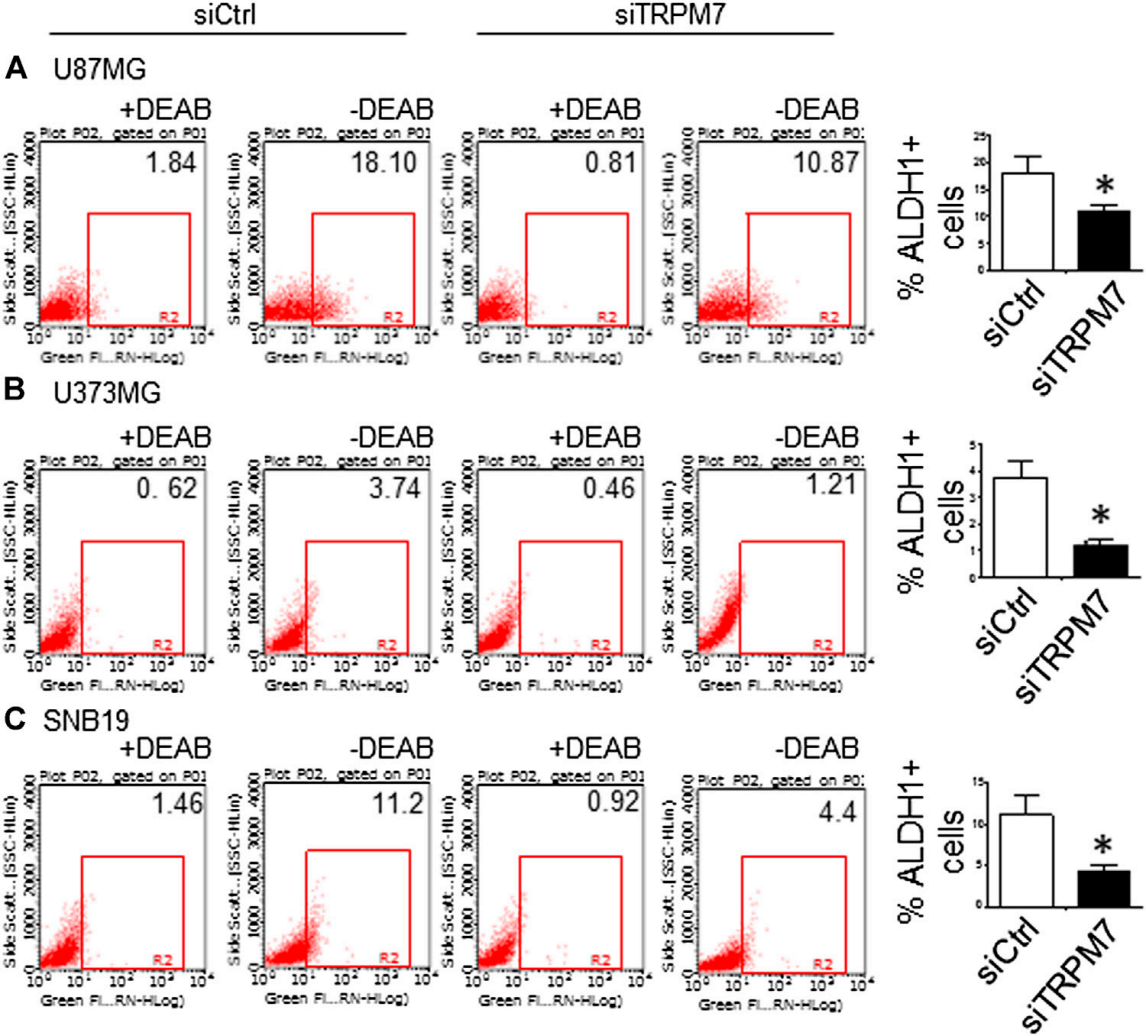
GSC marker ALDH1 is positively correlated with TRPM7 expression in glioma cells. **(A–C)** ALDH1 enzymatic activities determined by the Aldefluor assay was performed in TRPM7 - knockdown of A172 **(A)**, U373MG **(B)**, and SNB19 **(C)** glioma cell lines.

**FIGURE 7:**
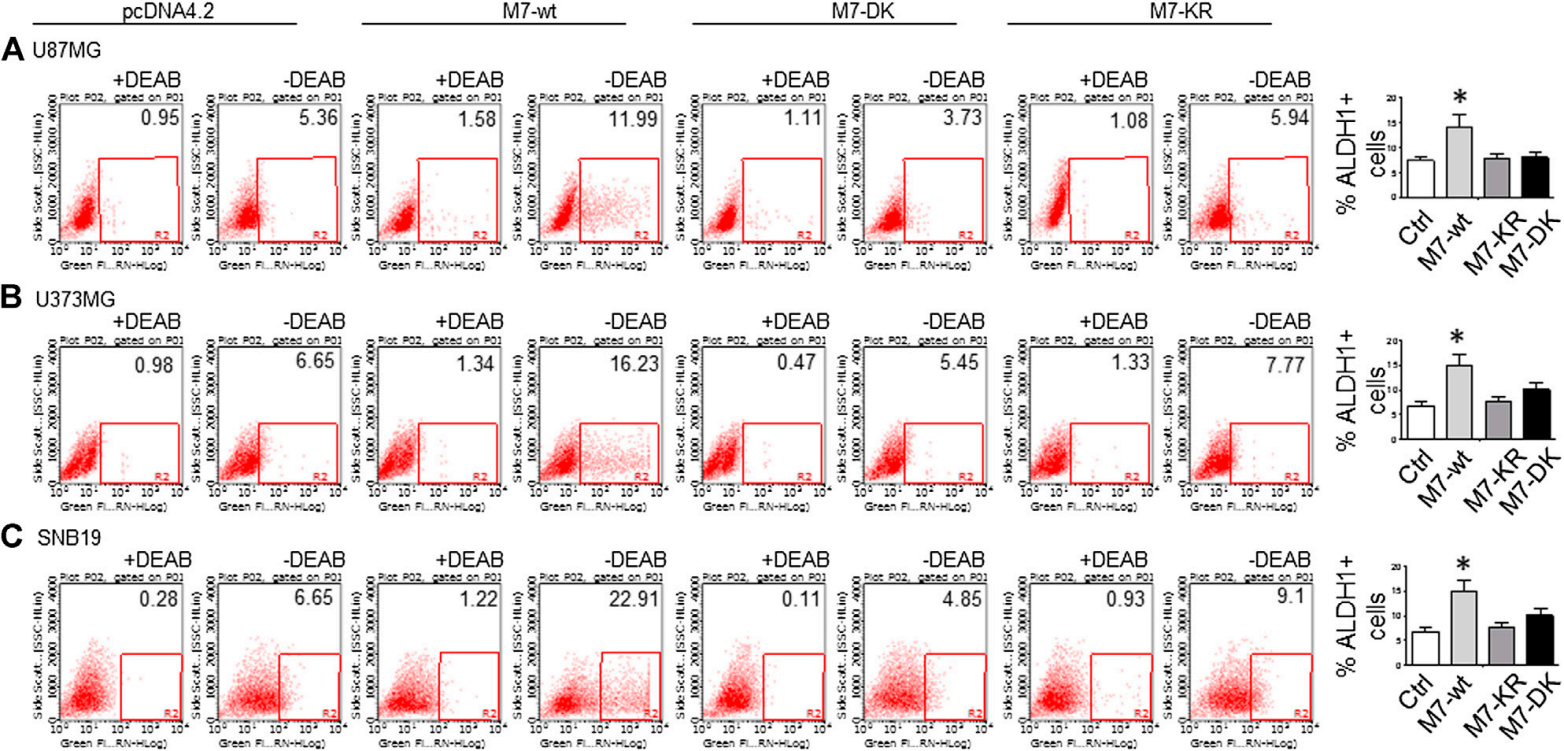
**(A–C)** ALDH1 enzymatic activities determined by the Aldefluor assay was performed in A172 **(A)**, U373MG **(B)**, and SNB19 **(C)** glioma cells transfected with wild-type human TRPM7 (M7-wt) and two α-kinase-inactive mutants ( M7-KR or M7-DK) along with controls. A comparison of the percentage of ALDH1 (+) in the above cells is shown as bar graphs representing mean ± S.D. to the right to each image.

**FIGURE 8:**
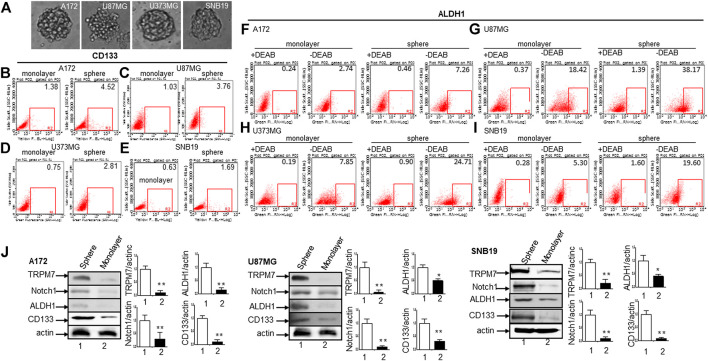
The cancer stem cells from glioma expressing high levels of glioma stem cell markers CD133 and ALDH1 along with TRPM7 and Notch1. **(A)** Morphologies of serum-free medium (SFM)-cultured stem-like tumorspheres from A172, U87MG, U373MG, and SNB19 glioma cells. **(B–E)** Flow cytometric analysis to measure CD133 expression in cells grown in monolayer and stem-like tumorspheres of A172 **(B)**, U87MG **(C)**, U373MG **(D)**, and SNB19 **(E)**. **(F–I)** ALDH1 activities determined by the Aldefluor assay in cells grown in monolayer and stem-like tumorsphere of A172 **(F)**, U87MG **(G)**, U373MG **(H)**, and SNB19 **(I)**. **(J)** The protein expression of TRPM7, Notch1, CD133, and ALDH1 determined by immunoblot and densitometry analysis with a fold change of each protein after normalization to individual loading control β-actin **(J)**.

(1) Inhibition of TRPM7 down-regulates Notch1 signaling. High-grade malignant gliomas are devastating, uniformly fatal tumors for which no effective therapies currently exist. GBM is the most common and aggressive primary brain tumor. We have reported that in the A172 cell line, TRPM7 channels, a subfamily member of the transient receptor potential (TRP), regulate GSC growth and proliferation through STAT3 and Notch1 signaling pathways (Liu et al., 2014). To further explore the mechanism that TRPM7 regulates Notch signaling pathway in the tumorigenesis of glioma, we examined all Notch receptor expressions in response to TRPM7 silencing in various glioma cell lines. To this end, the different glioma cell lines were transfected with either control siRNA (siCtrl) or TRPM7 siRNA (siTRPM7), followed by assaying the expressions of Notch1, Notch2, Notch3 and Notch 4 in each of the following cell lines: A172, U87MG, SNB19, and U373MG. At the mRNA levels, TRPM7 silencing increases Notch 4 mRNA expression in A172, increases Notch 2 mRNA expression in U87MG, decreases Notch 2, 3, and 4 mRNA expression in SNB19, and increases Notch 3 and Notch 4 mRNA in U373MG (Supplementary Figure 1). At the protein levels, we first determined whether or not TRPM7 protein is expressed in these glioma cell lines. As shown by the Western blot, all glioma cell lines A172, U87MG, U373MG, SF767, and SNB19, as well as two cell lines resistant to TMZ, T98G, and LN18, express an almost equal amount of TRPM7 with U87MG and SNB19 having slightly lower levels of expression (Figure 3A). The data show that in A172 cells, upon TRPM7 downregulation, the active form of Notch1, the intracellular domains of Notch1 (NICD), expression along with Notch1 target genes Hey2 and Survivin were decreased (Notch activity is often measured by the expression levels of its direct target genes). Expression of full length (FL) of Notch2 was found to be decreased, while Notch4 was increased (Figure 3B). A172 and U87MG both have wild-type *TP53*, *PTEN* mutations, and *p14* ^*ARF*^/p16 deletion (Ishii et al., 1999). However, U87MG cells express high levels of VEGF as compared to A172 expressing high levels of bFGF. Despite the difference in growth factors secretion, identical expression patterns of Notch signaling pathway activation were observed in U87MG cells compared to the A172; Notch1, Hey2, Survivin, and Notch3 NICD decreased, while the full length of Notch4 increased (Figure 3C). SNB19 and U373MG (Figure 4) have mutated *PTEN* (Memmel et al., 2014; Chakrabarti and Ray, 2015), and share common origins; however, these two cell lines have evolved to exhibit distinct karyotypes and drug sensitivities (Stepanenko and Kavsan, 2014). In SNB19, Notch1 NICD, Notch3 NICD, and Hey2, Survivin decrease as siTRPM7 downregulated TRPM7 with Notch1 FL increase whereas Notch2 NICD and Notch4 NICD did not change (Figure 3D). Interestingly, all three cell lines demonstrate reduced Notch1 and the target genes Hey2 and Survivin expression consistently when the TRPM7 gene was silenced, implying that the decreased expression of TRPM7 is correlated with decreased Notch active component Notch1 NICD in all glioma cell lines tested. Therefore, our results indicate that TRPM7 regulates the Notch1 pathway in all glioma cell lines even though gliomas are highly heterogeneous with variation in biological characteristics among different glioma cell lines. Moreover, the discrepancy between Notch mRNA (Supplementary Figure 1) and protein expression indicates that the decreased level of active forms NICD in response to siTRPM7 may not be caused by reduced mRNA.
(2) The upregulation of TRPM7 increases Notch1 signaling. To further investigate the correlation between TRPM7 and Notch receptors, we transfected U87MG cells with (a) wild-type human TRPM7 (wtTRPM7 or M7-wt); (b) constructs that rendered TRPM7 inactive with a point mutation in the ATP binding site of the α-kinase domain (K1648R, or M7-KR) or the α-kinase domain was deleted (Δkinase or M7-DK) as described in our previous publication (Wan et al., 2019). We decided U87MG as the cell line to investigate the effect of TRPM7 overexpression on Notch1 activation because the endogenous expression of TRPM7 is lowest among all cell lines tested (see Figure 3A). The transfection efficiency was first determined by Western blot. U87MG, transfected with M7-wt, M7-KR, and M7-DK constructs, expressed high TRPM7 protein levels compared to that of the control, and this indicates the high transfection efficiency of the system (Figure 4A). Considering both TRPM7 and Notch signaling function as oncogenes in glioma formation, it is not surprising to see that U87MG cells, transfected with M7-wt, express increased levels of Notch1 NICD, Notch2 NICD, and Notch3 NICD. By contrast, U87MG cells, transfected with kinase mutants M7-KR and M7-DK (Figure 4A), express decreased levels of Notch1 NICD, Notch2 NICD compared with its wild-type partner, indicating TRPM7 with unfunctional kinase activity negatively regulates Notch1 and Notch2 activities. Interestingly, when cells harbor kinase-mutants, their Notch3 NICD are higher, implying a probable feedback regulation by unknown factors due to loss of TRPM7 kinase activities. Notch4 NICD is not significantly affected by the introduction of neither M7-wt nor the two kinase domain mutants. Our results suggest that Notch1 and Notch2 activities positively correlate with TRPM7 channel activities, where the activities of Notch1 and Notch2 are partly determined by TRPM7 kinase activity. Therefore, our results show that TRPM7 regulates Notch signaling consistently through Notch1 signaling in glioma tumorigenesis.
(3) To further confirm that TRPM7 regulates Notch signaling, HEK-293 cells, a cell line derived from human embryonic kidney cells, were transfected with a pcDNA4/TO plasmid that allowed tetracycline-inducible protein expression of wild-type of hTRPM7 (HEK293: TRPM7). In brief, when cells were treated with 1 μg/ml of tetracycline, TRPM7 protein expression was induced by tetracycline-controlled transcription (Nadler et al., 2001; Jiang et al., 2007). As detected by Western blot in Figure 4B, TRPM7 was induced to express in HEK293 cells, either as an exogenous HA-tagged protein or a combination of endogenous and exogenous TRPM7 protein (Figure 4B) in the presence of an appropriate tetracycline concentration. The results show that Notch1, 2, and 4 NICD, Notch target gene Hey2 and Survivin, as well as GSC markers ALDH1 and CD133 are downregulated (Figure 4B) in response to increased TRPM7 expression. It is understandable that the changed pattern in Notch Signaling in HEK-293 is different from that of glioma cell lines; This is because of the distinctiveness of normal and tumor cells' biological behaviors. However, this data from the non-tumor cell line, from another point of view, further confirm that TRPM7 is involved in Notch signaling pathway regulation.
(4) GSC markers CD133 and ALDH1 are correlated with TRPM7 in GBM. We have previously reported that TRPM7 positively regulates the GSC marker CD133 in A172 cells (Liu et al., 2014). To determine whether or not our previous findings are not only limited to A172 cells, we tested the number of CD133 + cells in response to changes in TRPM7 expressions in additional glioma cell (GC) lines. Consistent with our previous results, we found that once TRPM7 was knocked down by siRNA, the amount of CD133 + cells are decreased in A172 cells (Figure 5A), U373MG cells (Figure 5B), and SNB19 cells (Figure 5C). These results indicate that the downregulation of TRPM7 result in a reduced GSC population. When TRPM7 expression levels were increased by introducing M7-wt vectors into GC lines, the number of CD133 + cells increased in A172 (Figure 5D, left two panels), U373MG (Figure 5E, left two groups), and SNB19 cells (Figure 5F, left two panels). However, the kinase-dead mutants M7-KR and M7-KD fail to upregulate the number of CD133 + cells (Figures 5D–F, right two groups). In other words, if TRPM7 kinase domain was disrupted, TRPM7 activation would be affected, resulting in reduced expression of CD133, which is an indication of TRPM7 function loss due to unfunctional kinases. The data represent a representative experiment from three independent experiments performed in duplicate.
(5) To address the question of whether or not TRPM7 will significantly affect ALDH1, another GSC marker (Rasper et al., 2010; Liu et al., 2014; Sullivan et al., 2017), we chose a similar model of glioma cells as described above, in which TRPM7 is either underexpressed or overexpressed by siTRPM7 or TRPM7-related constructs. The ALDEFLUOR assay was conducted on all glioma cells. TRPM7 knockdown decreases the number of ALDH1-positive cells from 18.10 to 10.87% in U87MG cells (Figure 6A), from 3.74 to 1.21% in U373MG (Figure 6B), and from 11.2 to 4.4% in SNB19 cells (Figure 6C). Consistent with the relationship of TRPM7 and CD133, here, these results provide further evidence that TRPM7 silencing result in a reduced GSC population as characterized by the ALDH1+ population. On the contrary, overexpression of TRPM7 increases the ALDH1-positive cells in all of the three cell lines tested (Figures 7A–C). As occurred in CD133-positive cells, the kinase-dead mutants M7-KR and M7-KD fail to upregulate ALDH1+ cells (Figures 7A–C), which supports the concept of functional coupling between the TRPM7 channel and a kinase domain. In other words, if TRPM7 kinase domain was disrupted, TRPM7 activation would be affected, resulting in reduced expression of ALDH1, which is an indication of TRPM7 function loss due to unfunctional kinases. The data represent a representative experiment from three independent experiments performed in triplicate. The number of ALDH1- positive cells of the control with diethylaminobenzaldehyde (DEAB), a specific inhibitor of ALDH1, was used to confirm gating areas.
(6) Glioma neurospheres exhibit high levels of GSC markers CD133 and ALDH1 and express high levels of TRPM7 and Notch1. We placed the glioma cells in non-differentiating medium in non-adherent culture plates, and neurospheres were seen developing in all cell lines tested, including A172, U87MG, U373MG, and SNB19 (Figure 8A). As expected, GSC marker CD133-positive cells increased in GSC compared to glioma cells grown in monolayer: CD133-positive cells increased from 1.38 to 4.52% in A172 (Figure 8B), from 1.03 to 3.76% in U87MG (Figure 8C), from 0.75 to 2.81% in U373MG (Figure 8D), and from 0.63 to 1.69% in SNB19 (Figure 8E). In parallel, ALDH1-positive cells increased from 2.74 to 7.26 in A172 (Figure 8F), from 18.42 to 38.17% in U87MG (Figure 8G), from 7.85 to 24.71% in U373MG (Figure 8H), and from 5.30 to 19.0% in SNB19 (Figure 8I). To further determine the difference in TRPM7 and Notch1 expression in glioma cells grown in monolayer and glioma neurospheres, we compared those indexes between glioma cells grown in monolayer and GSC spheres derived glioma cells by performing Western blot analysis. As expected, TRPM7 and Notch1 are highly correlated with the GSC markers CD133 and ALDH1 in GSC spheres compared to that in the glioma monolayer cells (Figure 8J).

### Targeting Notch1 Suppresses Transient Receptor Potential Melastatin-Related 7-Induced Growth and Proliferation in Glioma Cells

**FIGURE 9:**
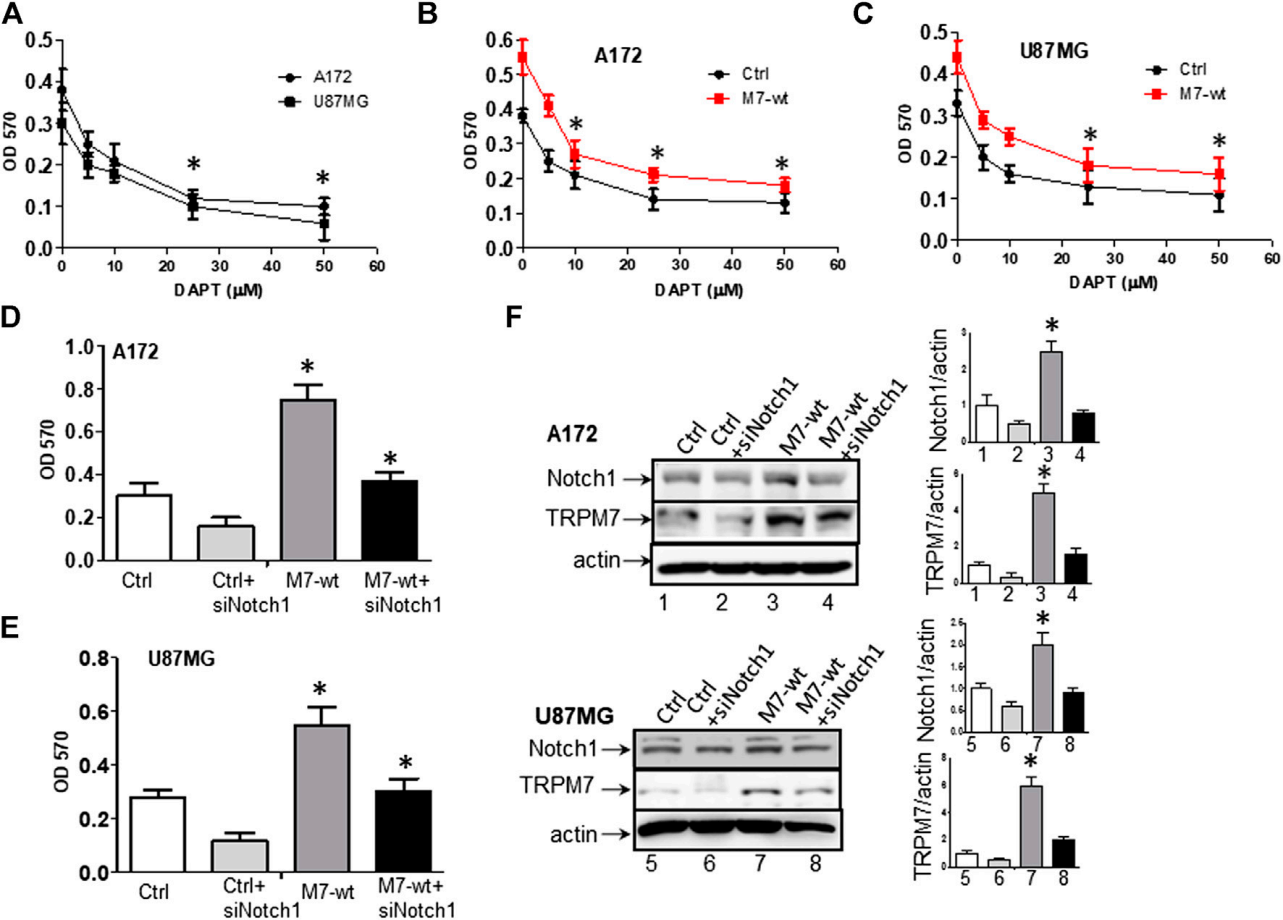
Targeting Notch1 suppresses TRPM7-induced growth and proliferation of glioma cells. **(A)** Dose-response curves obtained after 72 h treatment with 5, 10, 25, 50 µM DAPT of A172 and U87MG glioma cell lines grown in growth media. **(B)** Dose-response curves obtained after 72 h treatment with 5, 10, 25, 50 µM DAPT of TRPM7-overexpressed (M7-wt) A172 cells and control. **(C)** Dose-response curves obtained after 72 h treatment with 5, 10, 25, 50 µM DAPT of TRPM7-overexpressed (M7-wt) U87MG cells and control. One-way ANOVA revealed a statistically significant difference between TRPM7-overexpressed glioma cells and controls in both A172 and U87MG cells (*p < 0.05). **(D and E)** The growth rates obtained from A172 **(D)** and U87MG **(E)** cells at 72 h after transfection with TRPM7 (M7-wt), M7-wt along with siRNA Notch1 (siNotch1) and controls. **(F)** The transfection efficiency was detected by the expression of TRPM7 and Notch1 protein, as examined by Western blot. The corresponding densitometry analysis for Western blot was performed using the ImageQuant program, as shown in bar graphs on the right.

**FIGURE 10:**
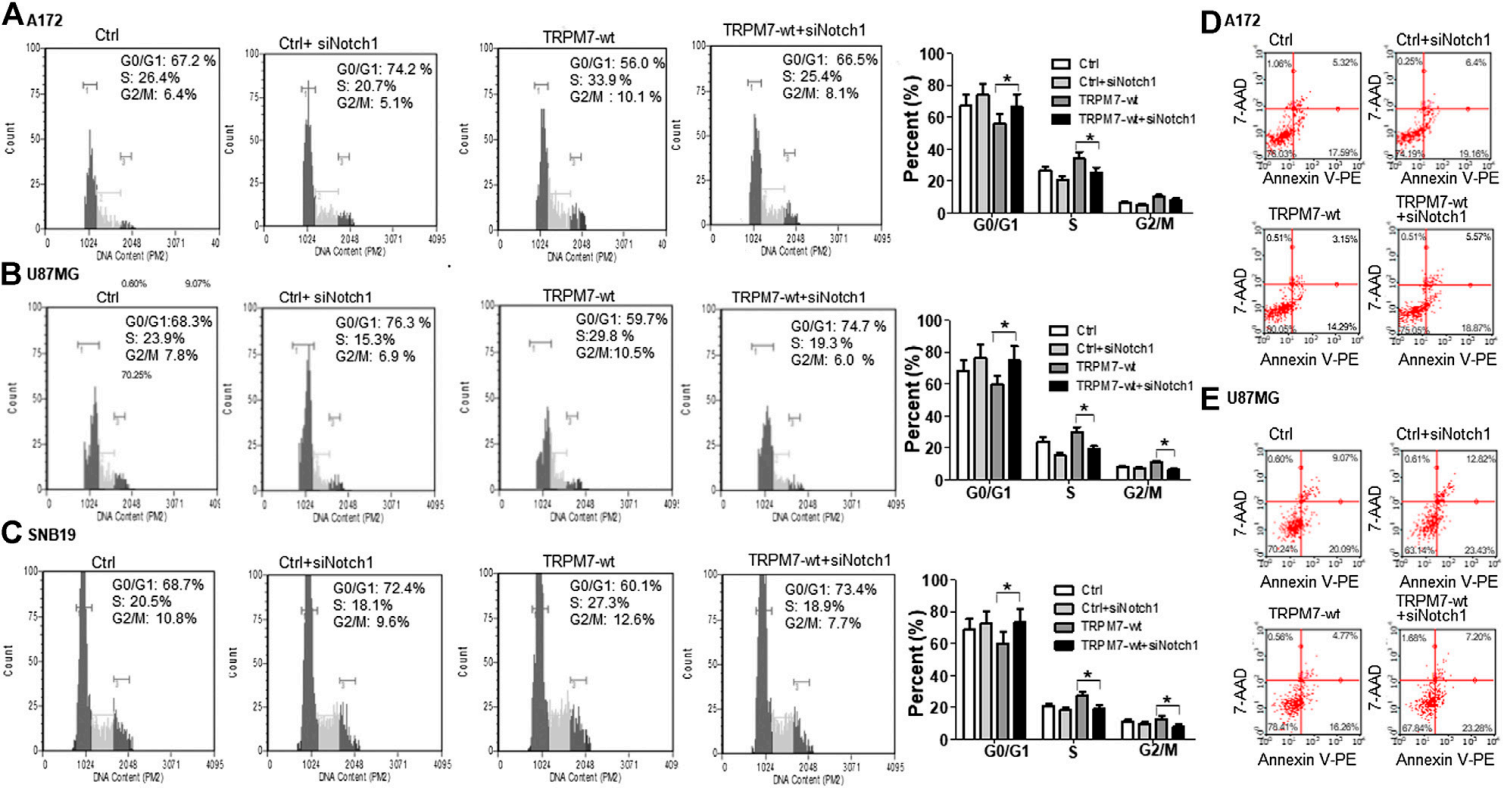
Targeting Notch1 suppresses TRPM7's effects on the growth and proliferation of glioma cells through G0/G1 arrest and induction of apoptosis. **(A–C)** Representative histogram plots of cell cycle distribution in A172 **(A)**, U87MG **(B)**, and SNB 19 **(C)** GBM cell line transfected with TRPM7 (M7-wt), M7-wt along with siRNA Notch1 (siNotch1) and controls, followed by staining with propidium iodide (PI) at 72 h and analyzed by flow cytometry. Downregulation of Notch1 reverses the TRPM7-induced deregulation of the cell cycle by decreasing the percentage of cells in the S and G2/M phase and increasing the percentage of cells in the G0/G1 phase. **(D and E)** Down-regulation of Notch1 increases the percentage of both early and late apoptotic cells labeled with Annexin V-PE/7-AAD of A172 **(D)** and U87MG **(E)** cells transfected with M7-wt, M7-wt along with siNotch1 and controls. Both early apoptosis in the lower right quadrant and late apoptosis in the upper right quadrant were taken into account because both are representative.

(1) Targeting Notch1 suppresses TRPM7-induced growth and proliferation in glioma cells. DAPT (2S)-N-[(3,5-difluorophenyl) acetyl)-L-alanyl]-2-phenyl] glycine 1,1-dimethylethyl ester), a typical Notch inhibitor, prevents full-length Notch1 from cleavage by the presenilin-γ-secretase complex to generate Notch1 NICD. Failure to release NICD into the nucleus results in the inactivation of Notch1 (Crawford and Roelink, 2007). As supported by our data above, Notch1 acts downstream to TRPM7 and contribute to the malignancy of glioma. Given this, we expect that the Notch1 signaling blockade will reverse the effects of TRPM7 on glioma proliferation. Therefore, first, we tested whether or not the inactivation of Notch1 results in deceased glioma cell proliferation. To this end, A172 and U87MG cells were selected for DAPT treatment from 5 to 50 µM for 72 h, as indicated in Figure 9A. The growth rates of both cell lines were inhibited by DAPT in a dose-dependent manner with IC50 about 25 µM. To strengthen the observation, the post-transfection A172 and U87MG cells with TRPM7 wild type constructs (M7-wt) along with controls were then treated with an increasing dose of DAPT for 72 h. As expected, TRPM7 promotes cell proliferation, which is dose-dependently decreased by DAPT in both A172 (Figure 9B) and U87MG (Figure 9C). To further support that the inhibition Notch1 reduces TRPM7-mediated cell viability, either small interfering Notch1 RNA (siNotch1) or controls were cotransfected with TRPM7-wt into A172 and U87MG cells. Using this model, we can easily see how genetically silencing Notch1 transcription affects TRPM7-mediated cell viability and growth. Our data show that the growth rates are significantly enhanced by the ectopic introduction of TRPM7 in both A172 (Figure 9D) and U87MG cells (Figure 9E) as compared to being significantly reduced by siNotch1 addition. The high transfection efficiency of both TRPM7-wt and siRNA Notch1 is depicted by Western blot assay in Figure 9F, where the TRPM7 expression is dramatically enhanced in cells transfected with M7-wt (lane 1 vs. Lane 3; lane 5 vs. lane 7), Notch1 NICD expression is remarkably inhibited by siNotch1 (lane 1 vs. lane 2; lane 3 vs. lane 4; lance 5 vs. lane 6; lane 7 vs. lane 8). Of note, when the two cell lines are treated with siNotch1, TRPM7 is downregulated (Figure 9F, lane 3 vs. lane 4; lane 7 vs. lane 8), suggesting that TRPM7 functions downstream of Notch1. Considering the data above, we believe there is mutual regulation between TRPM7 and Notch signaling.
(2) Targeting Notch1 compromises TRPM7-mediated changes in cell cycle progression. To further investigate the effects of Notch1 on TRPM7’s function, we investigated whether or not the inactivation of Notch1 could rescue the changes in cell cycle progression caused by TRPM7. To this end, A172, U87MG, and SNB19 glioma cells were cotransfected with siNotch1 and M7-wt for 72 h and then followed by cell cycle distribution analyzation with propidium iodide staining using flow cytometry to quantify the percentage of cells in different cell cycle phases. Inhibition of Notch1 activity for 72 h by siNotch1 induced a significant decrease in the percentage of cells in S and G2/M phase from 33.9 and 10.1% in A172 cells transfected with M7-wt to 25.4 and 8.1% in those transfected with M7-wt along with siNotch1. There is a concomitant increase in the percentage of cells in the G0/G1 phase (56.0–66.5%) (Figure 10A). These data indicated that when siNotch1 was added into the cell culture system, the increased effects on S and G2/M phase and decreased effects on G0/G1 phase by TRPM7 was compromised. In other works, Notch1 has replaced the TRPM7 function, or TRPM7 function through Notch1 signaling pathway. Similar to the results shown in A172 cells, siNotch1 also decreased in the percentage of cells in S and G2/M phase from 29.8 and 10.5% in U87MG cells transfected with M7-wt to 19.3 and 6.0% in those cotransfected with M7-wt and siNotch1; there was also an increase in G0/G1 phase from 59.7 to 74.7% (Figure 10B). A similar pattern was also observed in SNB 19 cells with decreased S (27.3–18.9%) and G2/M phases (12.6–7.7%) and increased G0/G1 phase (60.1–73.4%) when comparing the cells transfected with M7-wt alone to the cells cotransfected with M7-wt and siNotch1 (Figure 10C). These results indicate the downregulation of Notch1 abrogated the deregulated cell cycle distribution caused by TRPM7, in other words, downregulation of Notch1 compromised increased DNA synthesis and mitosis of tumor cells by TRPM7.
(3) Targeting Notch1 counteracts the role of TRPM7 in regulating apoptosis in glioma cells. A172 and U87MG glioma cells were treated with siNotch1 along with TRPM7 wild-type constructs for 72 h, followed by Annexin V and 7AAD binding assay. Results show that the percentage of apoptotic glioma cells increased from (17.59 + 5.32) to (19.16 + 6.4)% in the controls compared to those silenced by Notch1; and (14.29 + 3.15) to (18.87 + 5.57)% in TRPM7-wt transfected A172 cells compared to those co-transfected with TRPM7 and siNotch1. Similar to results shown in A172 cells, siNotch1 also induces apoptosis significantly from (20.09 + 9.07) to (23.43 + 12.82)% in the parental U87MG cells and (16.26 + 4.77) to (23.28 + 7.20)% in TRPM7-wt transfected U87MG cells (Figure 10D).

## Discussion

Major findings from the current study include: 1) TRPM7 mRNA expression is significantly increased in anaplastic astrocytoma, diffuse astrocytoma, and GBM patients compared to that in healthy brain tissue controls. 2) TRPM7's channel activity positively correlates with Notch1 activation and GSC markers CD133 and ALDH1. Downregulation of TRPM7 inactivates Notch1 signaling and its target genes as well as decreases the expression of CD133 and ALDH1, whereas the upregulation of TRPM7 activates Notch1 signaling, and increases the expression of CD133 and ALDH1. TRPM7's role in Notch1 activity, GSC marker CD133, and ALDH1 require functional coupling between TRPM7 channel and a kinase domain. 3) Targeting TRPM7 suppresses the growth and proliferation of glioma cells through G1/S arrests by decreasing the percentage of cells in S and G2/M phases and through glioma cell apoptosis. 4) Targeting Notch1 compromises the TRPM7-induced growth and proliferation of glioma cells.

TRPM7 was first described in 2001 and has been attracting increased attention from many fields due to its multifaceted functions. TRPM7 is involved in various cellular processes such as Ca^2+^, Mg^2+^, and Zn^2+^ homeostasis, mechanosensitivity, exocytosis, immune activation (Romagnani et al., 2017; Schappe et al., 2018), epigenetic modification (Krapivinsky et al., 2014), cell proliferation, migration, and differentiation. Consequentially, TRPM7 plays a causative role in tumorigenesis, ischemic diseases (both heart and brain), and neurodegenerative disorders (Chubanov et al., 2018). The unique molecular structure of TRPM7, which contains a transmembrane channel segment fused to a cytosolic α-type serine/threonine-protein kinase domain, highlights the functional complexity of this gene (Rios et al., 2020; Trapani and Wolf, 2020). TRPM7 is ubiquitously expressed in all mammalian cells examined so far, and it is well documented that the deletion of the entire *TRPM7* gene is lethal to mouse embryo at an early stage (Trapani and Wolf, 2020; Turlova et al., 2020). Furthermore, the elimination of the kinase domain is also fatal to mouse embryonic development (Ryazanova et al., 2010). It is accepted that TRPM7 signaling does not only depend on channel-mediated Mg^2+^ (Rios et al., 2020; Voringer et al., 2020) or Ca^2+^ influx (Song et al., 2020) but also on phosphorylation of downstream genes that require α-kinase (Voringer et al., 2020). Whether or not the α-kinase domain is required for TRPM7 channel-mediated ion influx remains controversial with no unanimous conclusion. Some groups reported that a functional α-kinase domain is required for TRPM7 channel activities (Ryazanova et al., 2004; Ryazanova et al., 2010; Song et al., 2017), others suggested that the α-kinase domain is not necessary for TRPM7 channel activity and functions in a TRPM7 channel-independent manner (Yogi et al., 2013; Romagnani et al., 2017). The latter was supported by that the specific inactivation of the TRPM7 α-kinase activity with a point mutation in the ATP binding site of the α-kinase domain (K1648R) did not contribute obvious pathologic phenotypes in homozygous TRPM7 kinase-dead mutant (*TRPM7* ^*R/R*^) mice (Kaitsuka et al., 2014). Recently, more mechanisms by which TRPM7's contribution to tumor formation have been explored. TRPM7 activates downstream targets annexin-1, calpain, and myosin, contributing to tumor cell migration and invasion. One of the newly found substrates of TRPM7 is RhoA. In hepatocellular carcinoma (HCC), TRPM7 activates RhoA, increases the cellular F-actin expression, increases myocardin-related transcription factors A and B (MRTFs) -Flaming A complex formation, therefore, facilitates MRTF nuclear localization and transcriptional activity that lead to cancer cell growth (Voringer et al., 2020). The epithelial-mesenchymal transition (EMT) is recognized to promote cancer cell invasiveness because of the high mobility and migratory abilities of mesenchymal cells once converted from carcinoma cells (Lo et al., 2017). Previously, TRPM7 was found to participate in EMT induced by tension (Kuipers et al., 2018) or EGF in breast cancer (Davis et al., 2014), and TGF-β in prostate cancer (Sun et al., 2018). Recently, Vanlaeys et al. provided convincing evidence that in mammary epithelial cells exposed to Cadmium (Cd^2+^), TRPM7 is induced and deregulates cytosolic Mg^2+^ balance and enhances cell invasiveness (Vanlaeys et al., 2020). To contribute the mechanism of TRPM7’s role in glioma, our current results first decipher that TRPM7 is responsible for sustained Notch1 signaling activation, enhanced expression of GSC markers CD133 and ALDH1, and regulation of glioma stemness, which contribute to malignant glioma cell growth and invasion. Interestingly, wtTRPM7 (M7-wt) increase the CD133 and ALDH1 expression in all glioma cell lines tested, however dead kinase domain of TRPM7 (M7-KR and M7-DK) downregulate the expression of CD133 and ALDH1 compared to controls (M7-wt), which indicates that disruption of kinase domain would reduce the TRPM7 activation resulting in reduced activation of stem cell markers CD133 and ALDH1. In other words, both TRPM7 channel and kinase activity of TRPM7 are required to fulfill its function in the regulation of GSC stemness (Figures 4, 6 and 7).

**TABLE 1:** List of primers used in the study.

Primer set	Forward 5′-3′	Reverse 5′-3′
TRPM7	CTT​TGA​CCA​AGA​GGG​AAT​GTG	GAC​CAA​GCG​ACC​ACA​AAA​AC
GAPDH	GAA​GGT​GAA​GGT​CGG​AGT​C	GAA​GAT​GGT​GAT​GGG​ATT​TC
Notch1	CACTGTGGGCGGGTC C	GTT GTATTGGTTCGGCACCAT
Notch2	AAT​CCC​TGA​CTC​CAG​AAC​G	TGG​TAG​ACC​AAG​TCT​GTG​ATG​AT
Notch3	GGC​TGT​GAA​CAA​CGT​GGA​AG	TGGCAAAGTGGTCCAACA
Notch4	TAGGGCTCCCCAGCTCTC	GGCAGGTGCCCCCATT

It is well accepted that Notch signaling activation contributes to human glioma tumorigenesis (Bazzoni and Bentivegna, 2019). Notch1 expression and activation contribute to glial cell transformation and glioma growth/survival, migration/invasion through Ras (Kanamori et al., 2007), β-catenin, NF-κB, AKT activation and downregulation of PTEN (Zhang et al., 2012; El Hindy et al., 2013). Recently, Ulasov et al. found that Temozolomide (TMZ) facilitates nuclear translocation of MMP14, followed by extracellular release of canonical Notch1 and Notch3 ligand Dll4, which in turn promotes cleavage of Notch3 and its nuclear translocation and induces glioma sphering ability and stemness (Ulasov et al., 2020). Notch4 is proposed as a less differentiated marker for glioma cells, and Notch4 expression increases from low-grade astrocytoma to high-graded GBM (El Hindy et al., 2013; Dell'albani et al., 2014). The upregulation of Notch ligand JAG1 and targets Hey1, Hey2, Hes1 in brain tissues of glioma patients compared to that of healthy brain tissues also highlight the Notch signaling pathway as a potential therapeutic target in glioma patients (El Hindy et al., 2013). The cross interaction among Notch and other pathways, such as Hedgehog (Chang and Lai, 2019) and EGF-related pathways (Cristofaro et al., 2020), implicates the molecular targets with overlapping functions should be prioritized as therapeutic targets. As more in-depth research developed, the contribution of Notch signaling in glioma progression is extended to non-canonical Notch ligand 1 (DLK1). DLK1 is a transmembrane protein that can be cleaved by ADAM17 and translocates to the nucleus in glioma cells under hypoxic conditions, leading to p53 and PI3K pathway activation (Grassi et al., 2020). Most recently, Otani provided convincing evidence that oncolytic HSV-infected glioma cells activate Notch signaling in adjacent tumor cells, which sensitizes tumors to gamma-secretase inhibition (Otani et al., 2020). In the current study, we first show that TRPM7-mediated Notch1 signaling activation is a crucial contributor to glioma cell proliferation and GSC stemness. TRPM7 increases the growth and proliferation of glioma cells through unlocking G1/S arrests, stimulating cell entry into S and G2/M phases, and inhibiting glioma cell apoptosis (Figure 2 and Figure 9). Silencing of Notch1 can revert the effects of TRPM7 on cell cycle and programmed cell death in glioma cells (Figure 10), which indicates that Notch1 can replace the TRPM7 function, or TRPM7 functions through Notch1 signaling pathway.

CD133 serves as a GSC marker and has long been used to sort out GSC for further *in vitro* and *in vivo* stem-like characteristics studies (Pollard et al., 2009; Logun et al., 2019; McAbee et al., 2019). ALDH1 is identified as another biomarker for GBM; high levels of ALDH1 usually indicate high invasive features and resistance to EGFR inhibition (Smith et al., 2017; McKinney et al., 2019), characteristics that are used as a measure of stem-like properties for GBM. A recent meta-analysis showed that high expression of ALDH1 is associated with a high WHO grade of glioma and a worse prognosis in glioma patients (Wang et al., 2017). Our data show that TRPM7 expression highly correlates with the expression of CD133 and ALDH1 (Figure 8) in both bulk glioma cells and in GSCs (Figure 8), which indicates that TRPM7 is very important in GSC formation.

High heterogenous GBM contains GSC and non-stem tumor cells as well as other non-tumor cells, where GSC interreact with other cells in the microenvironment can further potentiate the malignancy of GBM (Tao et al., 2020). Targeting GSCs and their interactions with other components may serve as the strategy for current GBM therapy (Kierulf-Vieira et al., 2020). In summary, TRPM7 is responsible for sustained Notch1 signaling activation, enhanced expression of GSC markers, and regulation of glioma stemness, all of which contribute to malignant glioma cell growth and invasion. For the prospects, several siRNA may be combined; for instance, two siRNAs targeting TRPM7 and Notch1 could be encapsulated together in a single nanoparticle to obtain synergistic therapeutic effects.

## Data Availability Statement

The raw data supporting the conclusions of this article will be made available by the authors, without undue reservation.

## Author Contributions

SG and ML designed the study protocol. JW, AG, PK, and TS performed experiments based on glioma cell cultures and evaluated the data with the help of ML. ML performed the biostatistical evaluation of the data. AG and ML wrote the manuscript with contributions and final approval by all authors. SG and YJ contributed to the critical reading and revision of the manuscript.

## Funding

This study was supported by the SC3 grant from NIH NIGMS GM121230 to ML and partly supported by the Morehouse School of Medicine Tx Pilot grant. The funding bodies had no role in the design, collection, analysis, and interpretation of the study’s data and in writing the manuscript.

## Conflict of Interest

The authors declare that the research was conducted in the absence of any commercial or financial relationships that could be construed as a potential conflict of interest.
