# Supplementary material for: TRPM7 Induces Tumorigenesis and Stemness Through Notch Activation in Glioma
Source: Front Pharmacol. 2020 Dec 14;11:590723. doi: 10.3389/fphar.2020.590723 (PMC7768084; doi:10.3389/fphar.2020.590723)
Supplement: Supplementary file 1 [file presentation1.pdf]

Supplementary information

Real-time RT-PCR analysis: Total RNA isolation, cDNA synthesis, and PCR amplification were performed as previously described (Liu et al., 2013). Cell pellets were stored in Trizol reagent and homogenized in fresh Trizol. Total RNA was isolated from cells using a miRNeasy Kit (Qiagen, Valencia, CA) and quantified using the Nanodrop N-1000 by Agilent Biosystems (Santa Clara, CA). Purified total RNA (0.75 µg) was reverse transcribed using the iScript cDNA Synthesis Kit according to the manufacturer's protocol (Bio-Rad Laboratories, Inc, Hercules, CA). Reverse transcription was performed by using random hexamers at 25°C for 5 min, 42°C for 30 min, and 85°C for 5 min. After diluting ten times, the cDNA was then amplified using iQ SYBR Green Supermix (Bio-Rad Laboratories, Inc.) according to the manufacturer's protocol under the following conditions: activation of the Taq DNA polymerase at 95°C for 3 min, 40 cycles at 95°C for 10 s (denaturation), and 61°C for 45 s (combined annealing and extension). The quantitative gene analysis utilized the CFX Connect Real-Time PCR Detection System. Each condition was conducted in biological triplicates, and each biological replicate was amplified in technical triplicates. Relative expression for each gene was evaluated using the $2^{-\Delta\Delta Ct}$ Livak method, and GAPDH was used as the reference gene (Haddock et al., 2019). We used the melting curve analysis to assess whether or not the intercalating dye qPCR assays produced single, specific products. The single peak was observed for each specific gene, which represented as a pure single amplicon, indicating the specificity of each primer for each specific gene. The primers used in the study was listed in Table 1.

Supplemental, Fig 1. TRPM7 mRNA is expressed in GBM and correlates with Notch1 activity - Inhibition of TRPM7 downregulates Notch1 signaling. All Notch receptor Notch1, Notch2,

Notch3, and Notch4 mRNA expressions were examined in response to TRPM7 silencing in various glioma cell lines A172 (A), U87MG (B), SNB19 (C) and U373MG (D) after they were transfected with either control siRNA (siCtrl) or TRPM7 siRNA (siTRPM7) followed by assaying mRNA expressions using quantitative PCR.
