# Supplementary material for: TRPM7 Induces Tumorigenesis and Stemness Through Notch Activation in Glioma
Source: Front Pharmacol. 2020 Dec 14;11:590723. doi: 10.3389/fphar.2020.590723 (PMC7768084; doi:10.3389/fphar.2020.590723)
Supplement: Supplementary file 2 [file presentation2.pptx]

## Slide 1
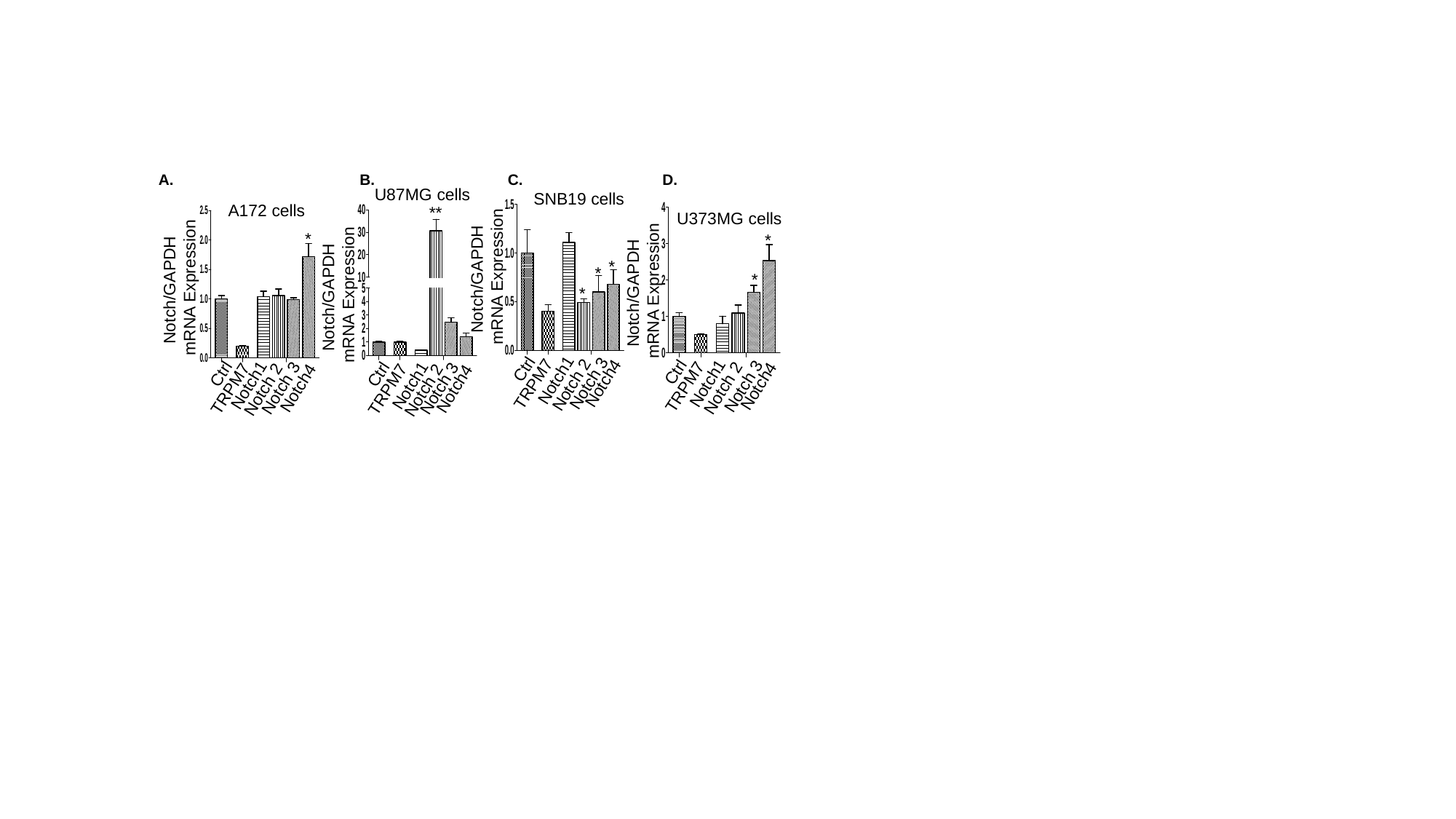

A.
B.
C.
D.
U87MG cells
SNB19 cells
A172 cells
**
U373MG cells
*
*
*
*
Notch/GAPDH
 mRNA Expression
*
Ctrl
Notch1
Notch4
Notch 3
Notch 2
TRPM7
Ctrl
Notch1
Notch4
Notch 3
Notch 2
TRPM7
Ctrl
Notch1
Notch4
Notch 3
Notch 2
TRPM7
Ctrl
Notch1
Notch4
Notch 3
Notch 2
TRPM7
Notch/GAPDH
 mRNA Expression
Notch/GAPDH
 mRNA Expression
*
Notch/GAPDH
 mRNA Expression
